# Ultraviolet Light (UV) Inactivation of Porcine Parvovirus in Liquid Plasma and Effect of UV Irradiated Spray Dried Porcine Plasma on Performance of Weaned Pigs

**DOI:** 10.1371/journal.pone.0133008

**Published:** 2015-07-14

**Authors:** Javier Polo, Carmen Rodríguez, Jesús Ródenas, Louis E. Russell, Joy M. Campbell, Joe D. Crenshaw, David Torrallardona, Joan Pujols

**Affiliations:** 1 APC EUROPE, S.A. Avda. Sant Julià 246-258. Pol. Ind. El Congost. E-08403 Granollers, Spain; 2 APC Inc. 2425 SE Oak Tree Court, Ankeny, IA 50021, United States of America; 3 Monogastric Nutrition, Institut de Recerca i Tecnologia Agroalimentàries (IRTA), Mas de Bover, Ctra. Reus—El Morell, km 3.8, 43120 Constantí, Spain; 4 Centre de Recerca en Sanitat Animal (CReSA)—Institut de Recerca i Tecnologia Agroalimentàries (IRTA), Campus UAB, 08193 Bellaterra, Barcelona, Spain; Virginia Polytechnic Institute and State University, UNITED STATES

## Abstract

A novel ultraviolet light irradiation (UV-C, 254 nm) process was designed as an additional safety feature for manufacturing of spray dried porcine plasma (SDPP). In Exp. 1, three 10-L batches of bovine plasma were inoculated with 10^5.2±0.12^ tissue culture infectious dose 50 (TCID_50_) of porcine parvovirus (PPV) per mL of plasma and subjected to UV-C ranging from 0 to 9180 J/L. No viable PPV was detected in bovine plasma by micro-titer assay in SK6 cell culture after UV-C at 2295 J/L. In Exp. 2, porcine plasma was subjected to UV-C (3672 J/L), then spray dried and mixed in complete mash diets. Diets were a control without SDPP (Control), UV-C SDPP either at 3% (UVSDPP3) or 6% (UVSDPP6) and non-UV-C SDPP at 3% (SDPP3) or 6% (SDPP6). Diets were fed ad libitum to 320 weaned pigs (26 d of age; 16 pens/diet; 4 pigs/pen) for 14 d after weaning and a common diet was fed d 15 to 28. During d 0 to 14, pigs fed UVSDPP3, UVSDPP6, or SDPP6 had higher (*P* < 0.05) weight gain and feed intake than control. During d 0 to 28, pigs fed UVSDPP3 and UVSDPP6 had higher (*P* < 0.05) weight gain and feed intake than control and SDPP3, and SDPP6 had higher (*P* < 0.05) feed intake than control. Also, pigs fed UVSDPP had higher (*P* < 0.05) weight gain than pigs fed SDPP. In conclusion, UV-C inactivated PPV in liquid plasma and UVSDPP used in pig feed had no detrimental effects on pig performance.

## Introduction

Spray dried porcine plasma (SDPP) is a dehydrated product obtained from blood of healthy pigs collected at slaughterhouses. Spray dried porcine plasma is a protein source used in pig feed that has many functional components that significantly improves pig performance [1,2]. At a manufacturing plant, plasma is separated from red blood cells by centrifugation, concentrated and submitted to a thermal process of >80°C throughout its substance. Although SDPP is a safe product, the introduction of redundant safety features to the manufacturing process is prudent for further assuring safety as microbes or viruses may evolve over time and adaption of redundant safety features will be aligned with suggested recommendations from international agencies to minimize potential risk derived from the use of biological products [3]. Having a proven and well recognized redundant safety feature with broad spectrum lethality against pathogens ranging from small viruses to protozoa will also provide added customer assurance that SDPP is safe to use in animal feed.

Ultraviolet wavelength at 254 nm (UV-C) is a non-thermal process that disrupts cellular transcription and replication leading to death of bacteria, viruses, and molds [4] and is used for industrial disinfection of water, milk, and fruit juice with limited negative effects on nutritional quality of the treated liquids [5,6]. However, for large industrial volumes of turbid liquids, such as animal plasma, a special UV-C designed process is required to assure that the liquid has a turbulent flow regime through a thin chamber that thoroughly exposes the liquid surface area to UV-C. Furthermore, this uniquely designed UV-C system should not damage the functional components attributed to the beneficial performance effects associated with spray dried plasma in animal feed.

The objectives for these experiments were to evaluate the efficacy of this uniquely designed UV-C process to inactivate porcine parvovirus (PPV) inoculated in liquid bovine plasma. We selected PPV because it is recognized as a model for heat and chemical resistant viruses [3], and therefore could potentially be more resistant to the effects of UV-C. Also, because spray dried plasma contains functional components that have beneficial effects on pig performance, it was prudent to compare performance of weaned pigs fed diets with UV-C processed SDPP compared to regular SDPP to assure that the UV-C process did not result in damage to the functional components in SDPP.

## Materials and Methods

### Ethic Statement

Pigs used in experiment 2 were housed in compliance with the Directive 2010/63/EU of the European parliament and of the council of 22 September 2010 on the protection of animals used for scientific purposes. The animal experimental procedure was approved by the Animal Experimentation Ethics Committee of IRTA and no specific permit was required. The zootechnical procedures used in this experiment (i.e. weighing) were carried out by experienced and authorized personnel and were not susceptible for causing suffering to the animals.

### Novel pilot-scale UV-C system

The UV-C reactor system was designed and manufactured by Sure Pure Operation AG (Zug, Switzerland). A reactor typically consists of a stainless steel inlet and outlet chamber with a stainless steel corrugated spiral tube between the chambers. Stainless steel used was 316 grade. Inside the spiral tube is an UV-C 254 nm wavelength germicidal lamp of 100 Watt (W) output (30 W UV-C output) which is protected by a quartz sleeve. The liquid flows between the corrugated spiral tube and the quartz sleeve. The tangential inlet of the reactor creates a high velocity and turbulence in the inlet chamber and brings the liquid into contact with the UV-C radiation. The liquid is pumped from the inlet chamber into the actual reactor, the gap between the quartz sleeve, and the corrugated spiral tubing at a minimum flow rate of 3800 L/h with a Reynolds value in excess of 7500, indicating turbulent flow.

The UV-C dosage will be expressed as J/L in this study. The operation time of the UV-C treatment is based on the quantity of product to be treated and the flow rate of the product feed. Only 9 s are required for 10 L of product to pass through the system once at a flow rate of 4000 L/h; thus, one pass of the product through the system is equivalent to a UV-C dose of 22.95 J/L or 23.40 mJ/cm^2^.

Manual sampling was done aseptically and liquid plasma was extracted directly from the flow stream. The temperature was continuously regulated by a thermometer installed adjacent to the lamps. A standard ‘Cleaning In Place’ (CIP) process as described by Keyser et al. [4] was implemented prior to and following each UV-C treatment to avoid microbial contamination.

### Experiment 1

A virus stock of PPV (NADL-2 strain, supplied by CRESA) was propagated in porcine kidney cell (SK) grown in modified Eagle medium supplemented with 10% tryptose phosphate broth, fetal bovine serum, and an antibiotic cocktail containing penicillin, streptomycin and amphotericin B. Fetal bovine serum used in the study was certified free of BVD, IBR and BRSV from the producer. In addition, it was tested for BVDV Ag by RT-PCR and antibodies by virus neutralization with negative results. Porcine parvovirus was propagated for 48 h in the SK6 cell line. The SK6 cells were kindly provided by Professor Albert Bosch of the enteric virus group in the Microbiology department of the Faculty of Biology at the Universitat de Barcelona. Supernatant was centrifuged and divided into 100-mL aliquots containing tissue culture infectious dose_50_ (TCID_50_) of 10^7.4^/mL. One hundred mL of stock virus was added to 10 L of liquid bovine plasma (~1.0% of total volume) to approximate 10^5.0^ TCID_50_/mL. Bovine plasma was derived from whole blood collected from a Spanish inspected slaughter facility (Mercabarna, Barcelona, Spain). Animals were inspected and approved to be slaughtered for human consumption. Blood was collected into stainless steel pans containing anticoagulant. Although the collection system was hygienic, potential environmental contamination could not be avoided and the collected blood was not sterile. After collection, blood was transported to the laboratory (APC Europe, S.A., Granollers, Spain) and plasma was separated by centrifugation. Bovine plasma was used to ensure Porcine parvovirus antibodies were not present. A 10-mL sample of the inoculated 10-L batch was frozen (–20°C) for later determination of viable viral particles. Three 10-L batches of plasma were subjected to UV-C using a pilot plant UV unit (SP-1 Unit, Sure Pure Operation AG, Zug, Switzerland) at a flow rate of 4000 L/h and irradiated through a 30 W UV-C output at 254 nm. Samples were collected after different UV dosages (0–9180 J/L) corresponding to different intervals of irradiation time (0, 5, 10, 15, 30, 45 and 60 min).

Samples of inoculated liquid plasma and irradiated plasma were frozen with dry ice prior to analysis of infectivity in SK6 cell cultures using the microtiter assay procedure [7].

To determine viral survival, 5 mL of each sample were inoculated on a 75 cm^2^ flask of SK6 cells. After three days, cell cultures were harvested and passed to new SK6 cell cultures. A total of three consecutive passages were made as a standard procedure to ensure that any viable virus had ample opportunity to adapt, grow, and multiply in the SK6 cell cultures. A tissue culture virus neutralizing (VN) test was performed to verify that the initial bovine plasma did not contain neutralizing antibodies to PPV. According to the method described by Remington [8], the theoretical limit of detection was estimated to be 0.23 viral particles per mL.

### Experiment 2

Porcine plasma was derived from whole blood collected from a Spanish inspected abattoir (Costa Brava, Girona, Spain). Animals were inspected and approved to be slaughtered for human consumption. Blood was collected into stainless steel pans containing anticoagulant. Blood was transported to the processing plant (APC Europe, S.A., Granollers, Spain) and plasma was separated by centrifugation. Ten 50-L batches of porcine plasma were divided in two pools of 25-L. One 25-L pool was irradiated using the UV system (SP-1 Unit, Sure Pure Operation AG, Zug, Switzerland) for 1 h at an average UV-C irradiation of 3672 J/L. The other 25-L pool (control) was maintained at 4°C without UV-C irradiation. Both pools of UV-C irradiated and control samples were dried using a pilot plant spray-drier (Anhydro Compact Spray Dryer, Anhydro A/S, Copenhagen, DK). Inlet temperature was 225 ± 3°C and outlet temperature was 81 ± 2°C. The suspension flow to the nozzle was 10.76 L/h. Airflow through the feeding nozzle was set at 500 m^3^ h^-1^ and air temperature was 20°C. The estimated dwell time was 30 s. From the 10 different batches, 35 kg of each SDPP were blended to obtain the composite UV-C irradiated spray-dried porcine plasma (UVSDPP) and control plasma (SDPP) for use in dietary treatments in a pig feeding study. The analyzed chemical and microbiological composition of the UVSDPP and SDPP is shown in Table 1.

**Table 1:** Chemical and microbial analyses of spray dried porcine plasma used in Experiment 2 (as-fed basis). Results are expressed as average±SD.

Item	UV-C Irradiated	Control
Specie of origin	Porcine	Porcine
Moisture (g/kg)	77.8±0.59	91.6±0.95
Protein (g/kg)	736±0.6	720±1.5
Ash (g/kg)	151±0.2	158±1.2
Insolubility (%)	1.88±0.68	2.65±1.40
Gel strength (N)	670±31.4	761±112
IgG (mg/g protein)	187±9.4	204±11
Microbial:		
Total aerobic plate count, cfu/g	< 10	8.3 x 10^4^±1.8 x 10^5^
Enterobacteriaceae, cfu /g	<10	<10
Salmonella in 25 g	Absence	Absence

The specie of origin was analyzed according to methods described by Polo et al. [9]. Moisture was analyzed following the AOAC procedure 930.15, and ash according to the Journal Officiel des Communnantés Européennes, Num L155/20. Crude protein was measured by the combustion method following the AOAC procedure 990.03. The agar radio immune diffusion method (RID) was used to analyze the amount of IgG in both SDPP [10]. Insolubility and gel strength were analyzed according to the methods described by Polo et al. [11]. For total aerobic plate count, Tryptone Soy Agar (Biokar Diagnostics, France) was used and the Enterobacteriaceae were identified using violet-red biliated glucose agar (VRBGA; Biokar Diagnostics, France). Salmonella was determined following the UNE EN ISO 6579:2003 method.

Two groups of 160 pigs ([Duroc x Landrace] x Pietrain; mixed sex) weaned at 26 d of age were used in a pig feeding performance study. Their average initial body weight was 8.13 ± 1.25 kg. For each weaning group, the pigs were housed in two rooms with 20 pens per room. The rooms were provided with automatic heating, forced ventilation, and completely slatted floors. Pigs were randomly distributed by initial body weight into sixteen blocks (8 blocks per weaning group; 4 blocks per room). Each block had five pens with four pigs per pen.

There were five experimental dietary treatments (Tables 2 and 3): a control diet based on wheat, barley, maize, soybean meal and 6% soy protein concentrate as a control protein source; two diets containing UV-C irradiated SDPP either at 3% (UVSDPP3) or 6% (UVSDPP6) and two diets containing non irradiated control SDPP at 3% (SDPP3) or 6% (SDPP6). The experimental diets (13.8 MJ ME; 13.5 g/kg lysine) were offered for a period of 14 d (pre-starter phase). During d 15 to 28, a common starter diet without SDPP was offered (13.6 MJ ME; 12.5 g/kg lysine). Feed was prepared in mash form and offered ad libitum. Feed and pigs were weighed at the start, and at d 14 and 28 (end of the experiment). Initial and final body weight, daily weight gain, feed intake, and gain to feed ratio were also measured or calculated.

**Table 2:** Composition of the diets used in Experiment 2, as-fed basis.

	Diets^1^			
Ingredients, g/kg	T-1	T-2 and T-3	T-4 and T-5	Starter
Wheat	25.00	25.00	25.00	-
Barley	20.00	20.00	20.00	38.8
Maize	15.00	15.00	15.00	25.0
Soybean meal,48% CP	13.14	13.86	14.33	22.6
Sweet milk whey	10.00	10.00	10.00	6.86
Soy protein concentrate	6.46	3.23	-	-
SDPP (source UV or C)	-	3.00	6.00	-
Wheat middling	3.00	3.00	3.00	-
Lard	3.50	3.50	3.50	3.27
Di-calcium phosphate	1.89	1.61	1.53	1.80
Calcium carbonate	0.39	0.56	0.60	0.31
L-Lysine-HCl	0.52	0.43	0.34	0.40
DL-Methionine	0.23	0.20	0.16	0.16
L-Threonine	0.20	0.15	0.09	0.14
L-Tryptophan	0.06	0.04	0.03	0.04
Salt	0.20	-	-	0.24
Ethoxyquin	0.02	0.02	0.02	0.02
Vit-Min complex^2^	0.40	0.40	0.40	0.40

^1^ T-1, control diet without SDPP; T-2, diet with 3% UVSDPP; T-3, diet with 3% control SDPP; T-4, diet with 6% UVSDPP; T-5, diet with 6% control SDPP. Diets were fed from d 0 to 14 post-weaning. All pigs were fed the common starter diet from d 15 to 28 post-weaning.

^2^Provided per kg of diet: vitamin A, 10000 IU; vitamin D_3,_ 2000 IU; vitamin E, 25 mg; vitamin B_1,_ 1.5 mg; vitamin B_2,_ 3.5 mg; vitamin B_6,_ 2.4 mg; vitamin B_12,_ 20 μg; vitamin K_3,_ 1.5 mg; calcium pantothenate, 14 mg; nicotinic acid, 20 mg; folic acid, 0.5 mg; biotin, 50 μg; choline, 300 mg; Fe, 120 mg; I, 0.75 mg; Co, 0.6 mg; Cu, 150 mg; Mn, 60 mg; Zn, 110 mg and Se, 0.37 mg.

**Table 3:** Calculated nutrient content of the diets used in Experiment 2, as fed basis.

	Diets			
Nutrients	T-1	T-2 and T-3	T-4 and T-5	Starter
Crude Protein	18.33	18.52	18.77	18.25
Crude Fibre	3.31	3.24	3.16	3.83
Fat	5.30	5.36	5.30	5.25
Ash	6.00	6.02	6.28	5.84
Lactose	7.29	7.29	7.29	5.00
Energy (MJ ME/kg)	13.8	13.8	13.8	13.6
Calcium	0.85	0.85	0.85	0.80
Phosphorous	0.70	0.70	0.73	0.70
Chloride	0.46	0.42	0.49	0.42
Sodium	0.15	0.19	0.30	0.15
Methionine	0.49	0.45	0.41	0.43
Methionine+Cystine	0.81	0.81	0.81	0.75
Lysine	1.35	1.35	1.35	1.25
Tryptophan	0.27	0.27	0.27	0.25
Threonine	0.88	0.88	0.88	0.81

^1^All pigs fed the common starter diet from d 15 to 28 of age.

### Statistical Analysis

Analysis of variance was performed using the GLM procedure of SAS (SAS Inst., Cary, NC). Average pen values were used as the experimental unit for the performance parameters. The model considered the effects of block and dietary treatment (5 diets). Data were adjusted by the covariant of initial body weight. Orthogonal contrasts were used to test the effects of SDPP processing (UV vs no UV) and dietary SDPP level (3% vs 6%). Results are presented as least squares means. The level of significance was set at *P* < 0.05 and trends were discussed at *P* < 0.10.

## Results

### Experiment 1: inactivation of PPV in liquid bovine plasma by the UV-C irradiation

**Fig 1:**
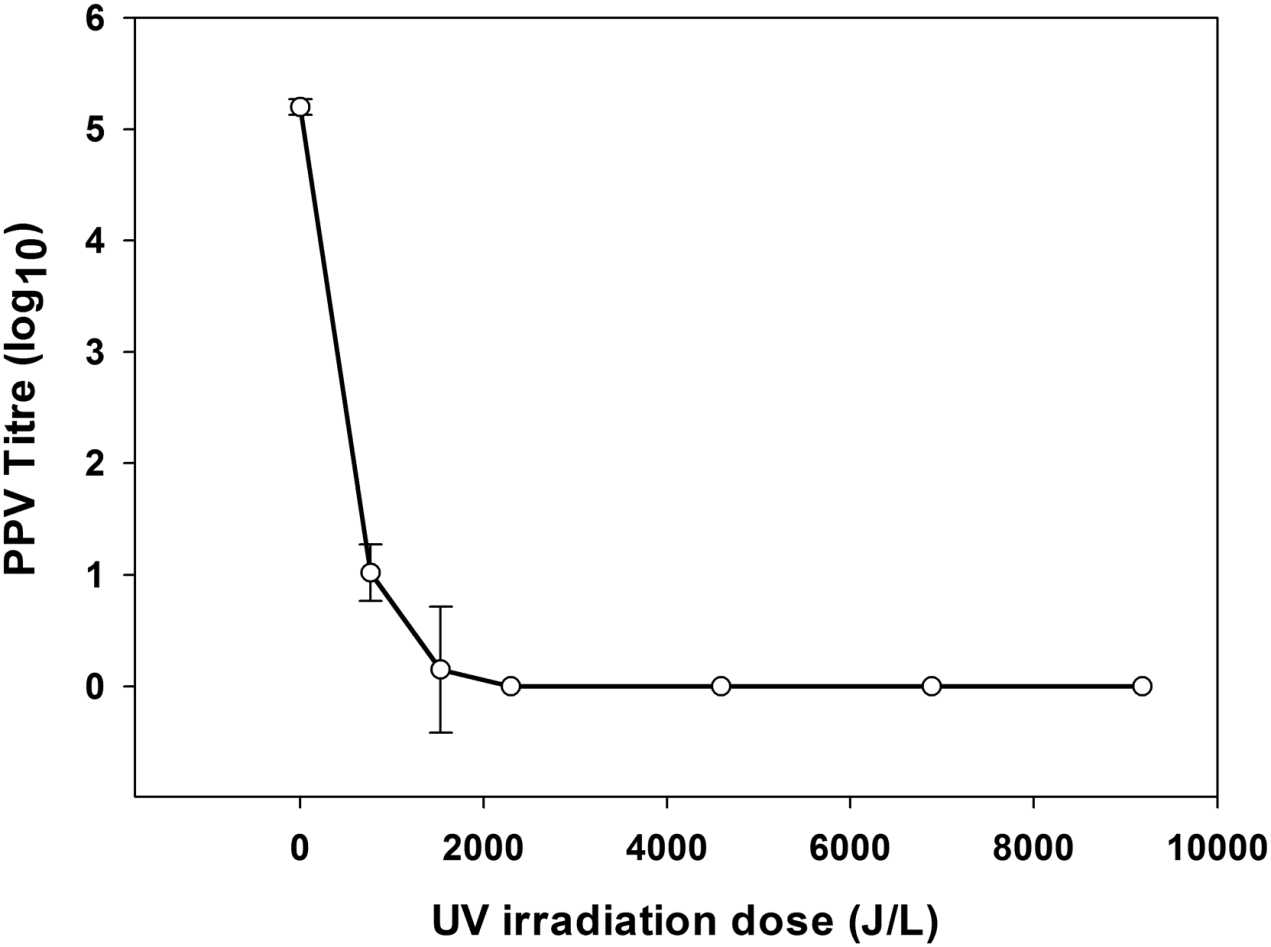
Porcine parvovirus virus inactivation in liquid bovine plasma after UV-C treatment. PPV titre expressed as log_10_; mean and SE; n = 3.

Neutralizing antibodies to PPV were not detected in the bovine plasma used in Exp. 1. After inoculation with PPV, the average infectivity of the three lots of liquid bovine plasma samples measured was 10^5.2±0.12^ TCID_50_/mL (Table 4, S1 and S2 Tables). Liquid bovine plasma samples contained no detectable viable PPV after 15 min of irradiation (accumulative UV-C irradiation of 2295 J/L; Fig 1, S1 and S2 Tables).

**Table 4:** Average Porcine Parvovirus (PPV) titres in inoculated bovine plasma used in Experiment 1 at each irradiation time (log10 values)^1^.

Time (min)	UV Irradiation Doses (J/l)	Mean of 3 replicates (log_10_ TCID_50_/mL^2^)	Confidence interval	Standard deviation
0	0	5.20	0.13341	0.12
5	765	1.02	0.50306	0.44
10	1,530	0.15	1.11065	0.98
15	2,295	Not detected^3^		
30	4,590	Not detected		
45	6,885	Not detected		
60	9,180	Not detected		

^1^Liquid bovine plasma samples were passed three consecutive times in SK6 cells for PPV to amplify any viable virus which may have been undetected in the first passage.

^2^Tissue culture infection dose was determined by microtiter assay procedure [7].

^3^The theoretical limit of detection of the method used was estimated to be 0.23 viral particles per mL.

Reduction factor was defined as log10 reduction between the quantity of virus detected in the initial material by unit of volume and the final quantity of virus after final treatment. Expected reduction factor at 5 min after UV-C exposure was 10^4.17±0.46^ TCID_50_/mL, and the expected reduction after 10 min was 10^5.05±0.99^ TCID_50_/mL. The probability of minimum virus titre after 15 min UV-C treatment was 0 (S3 Text).

### Experiment 2: performance of weaned pigs fed UV-C irradiated SDPP

The analysis of parameters comparing UV-C irradiated SDPP versus control SDPP indicated that both products had similar values for protein, ash, moisture, solubility, IgG content and gel strength (Table 1). Differences observed in these parameters were within the normal analytical variation of spray dried plasma. The variation between the UVSDPP and SDPP samples for protein, gel strength, IgG, and insolubility may be related to differences in final moisture content, as well as, differences associated with the 10 different 50 L batches of liquid plasma that were collected over different days. For example, natural variations of protein and IgG in the liquid plasma between days of collection are more common when small volumes are collected for experimental purposes. Under commercial conditions, liquid plasma from thousands of animals is pooled and this pooling effect minimizes variation of protein and IgG in the resulting spray dried product. However, there was a large reduction of total plate count for bacteria in the UVSDPP. These results indicated that UV-C irradiation had little effect on the physical-chemical parameters of SDPP, but it had a clear effect on reducing the bacterial contamination naturally present in commercial SDPP (Table 1).

During the pre-starter phase (d 0 to 14, Table 5), there were significant (*P* < 0.05) differences in weight gain and feed intake of pigs fed the various dietary treatments. Pigs fed diets with UVSDPP3 and UVSDPP6 or SDPP6 had higher (*P* < 0.05) weight gain and feed intake than the control group. The orthogonal contrasts revealed tendencies (*P* < 0.10) for greater weight gain and feed intake for the pigs fed UVSDPP than those fed SDPP, but no significant differences due to dietary SDPP level of inclusion.

**Table 5:** Least squares means of productive parameters of pigs^1^ fed diets with ultraviolet irradiated spray dried plasma (Experiment 2).

							Statistics		
	Control	UVSDPP-3%	SDPP-3%	UVSDPP-6%	SDPP-6%	Root MSE	Treatment	Source (UVSDPP vs SDPP)	Plasma Level (3% vs 6%)
Experimental pre-starter diets (d 0–14)									
BW d 0 (kg)	8.13	8.13	8.13	8.13	8.13	-	-	-	-
BW d 14 (kg)	10.84^c^	11.42^ab^	11.06^bc^	11.53^a^	11.31^ab^	0.581	*P* < 0.01	*P* < 0.1	NS^2^
ADG (g)	193^c^	234^ab^	210^bc^	243^a^	227^ab^	41.5	*P* < 0.01	*P* < 0.1	NS
ADFI (g)	284^b^	332^a^	309^ab^	348^a^	330^a^	46.7	*P* < 0.01	*P* < 0.1	NS
G:F ratio	0.69	0.70	0.71	0.71	0.69	0.059	NS	NS	NS
Common starter diets (d 15–28)									
BW d 28 (kg)	17.35^b^	18.10^a^	17.36^b^	18.10^a^	17.83^ab^	0.936	*P* < 0.05	*P* < 0.05	NS
ADG (g)	465	477	450	469	466	40.3	NS	NS	NS
ADFI (g)	723	756	728	759	750	53.1	NS	NS	NS
G:F ratio	0.65^a^	0.64^ab^	0.62^b^	0.62^b^	0.63^b^	0.031	*P* < 0.05	NS	NS
Cumulative data (d 0–28)									
ADG (g)	329^b^	356^a^	330^b^	356^a^	346^ab^	33.4	*P* < 0.05	*P* < 0.05	NS
ADFI (g)	510^c^	545^ab^	522^bc^	558^a^	544^ab^	44.7	*P* < 0.05	NS	NS
G:F ratio	0.67	0.66	0.65	0.65	0.65	0.029	NS	NS	NS

^1^Pigs (26 d old, 320 in total in two batches of 160 animals) were distributed in 16 blocks of 5 pens containing 4 pigs/pen (8 pens and 32 pigs per treatment in each batch). Pigs were fed their assigned experimental diets from d 0 to 14 and all pigs were fed a common diet from d 15 to 28 (BW: body weight; ADG: average daily gain; ADFI: average daily feed intake; G:F ratio: gain to feed ratio).

^2^NS *P* > 0.10

^abc^ Values in the same row with different letters are significantly different (*P* < 0.05).

During the starter phase (d 14 to 28, Table 5), in which all the pigs were fed a common diet, no differences due to the previous dietary treatment were observed for weight gain, or feed intake, but for feed efficiency (G:F) there was a tendency for improvement in the control group. At d 28, the pigs previously fed UVSDPP3 or UVSDPP6 continued having greater (*P* < 0.05) body weight than those from the control group. The orthogonal contrasts also revealed a higher (*P* < 0.05) final body weight for the pigs fed UVSDPP than those fed SDPP.

Over the whole trial (d 0–28, Table 5), pigs fed UVSDPP3, UVSDPP6 and SDPP6 had higher (*P* < 0.05) feed intake than control pigs and those fed UVSDPP3 and UVSDPP6 had higher (*P* < 0.05) body weight gain than those in the control and SDPP3 group. The orthogonal contrasts revealed a higher (*P* < 0.05) body weight gain for the pigs fed UVSDPP than those fed SDPP.

## Discussion

Spray dried porcine plasma is considered an inherently safe protein for use in animal feed, as evidenced by the fact that it was the first animal protein re-authorized in Europe for use in feed for animals intended for human consumption after the use of animal proteins in feed for meat animals was banned in 2001 due to the BSE crisis. During the manufacturing of spray dried blood products, there are several critical control points which reduce contamination and subsequent growth of pathogens in the initial collected blood. Spray-drying at high inlet and outlet temperatures is recognized as a “pasteurization” step to effectively inactivate different bacteria and viruses [12]. Spray-drying has been shown effective to inactivate coliform bacteria [13], as well as swine viruses, such as porcine reproductive and respiratory syndrome virus (PRRSV), pseudorabies virus [11], porcine epidemic diarrhea virus [14,15] and swine vesicular disease virus [16]. Likewise, feeding susceptible animals diets containing high inclusion levels of commercial SDPP for a feeding duration of 3 to 4 times higher than typically used in commercial practice, have shown that SDPP is a safe protein ingredient that does not transmit PPV and porcine circovirus type 2 (PCV2) [11,12,17,18], which are two of the more thermal and solvent resistant known viruses.

However, as microbes evolve and new pathogens are discovered, it is prudent to evaluate additional technology that may further minimize risks associated with feeding biological products to animals intended for human consumption. In case of animal plasma, there are limitations on additional safety features that can be applied without affecting the functionality of the plasma proteins. Thermal processing is well recognized to inactivate pathogens of concern in animal by-products [19], however, for plasma products, prolonged high temperature processing or chemical treatments denature proteins. Also, e-beam or gamma irradiation is not accepted by regulatory agencies in many countries or by some customers. Ultraviolet irradiation of different food and feed products has demonstrated pathogen inactivation efficacy without causing negative effects on the nutritional or physical qualities of the treated material [20,21] and it is widely recognized as a safe technology to use for inactivation of microbes in food or feed products.

Nevertheless, successful application of UV-C depends on the solids content and turbidity of the liquid, which can limit the penetration of UV-C light into the liquid and therefore affect efficacy [22]. Therefore, the application of a UV-C system to turbid liquids like animal plasma require a special design of the system to assure that the liquid has a turbulent flow regime and that the UV-C light is able to penetrate the liquid to be irradiated. The patented system (EP-1255444B1) has a turbulent flow (Reynolds number > 2800) that in conjunction with a very thin irradiation chamber allows the surface area of the turbid liquid to be exposed to UV-C. This system has been proven to inactivate microorganisms in turbid liquids like fruit juices and wine [4,23] and milk [24] and also is a system that is capable of processing large volumes of raw material that is common for the spray dried blood product business.

In experiment 1, UV-C irradiation was very effective for inactivating PPV, thus indicating successful reduction of a model virus inoculated in liquid bovine plasma. Also, UV-C irradiation has proved effective against different model viruses (human B19, PPV, simian virus 40, Sindbis virus, reovirus type 3, adenovirus type 5, murine parvovirus, encephalomyocarditis virus and bovine herpes virus type 2) and both Gram negative and Gram positive bacteria in human plasma products [25,26] and it is likely that this technology could be applicable to inactivation of emerging pathogens. Van Wyk and Gouws [27] also demonstrated the efficiency of UV irradiation to inactivate pathogenic and heat resistant microorganisms like *Bacillus* spp., *B*. *sporothermodurans*, *B*. *subtilis*, *E*. *coli* and *Salmonella* spp. in milk. In a literature review by Hijnen et al. [20], UV irradiation was reported to inactivate all waterborne viruses, bacteria and protozoa studied, and that the inactivation of microorganisms by UV could be described with first-order kinetics. In addition, Darnell et al. [28] demonstrated that UV light was effective against coronaviruses and recently Schoborg and Borel [29] have indicated that UV also inactivates porcine epidemic diarrhea virus (PEDV).

The results of the pig feeding experiment indicated that dietary SDPP improved pig performance (BW, ADG, ADFI and G:F) compared to pigs fed the control diet without SDPP and that UV-C SDPP tended to enhance performance in general over control SDPP. Previous research has focused on the effect of microbial quality of SDP on pig performance and the benefit of gamma irradiation, or the use of chemical products such as formaldehyde, to improve the bacterial quality of SDP and the performance of weaned pig [30–32]. DeRouchey et al. [30,31] in a series of experiments indicated that gamma irradiation of SDP, as a mechanism to reduce the bacterial count of plasma, could improve the growth of pigs fed diets containing this ingredient. The use of gamma irradiation is expensive and not approved in some countries or accepted by some customers from countries where this technology is permitted.

Chemical treatment of SDPP with formaldehyde is not allowed in many countries including the EU. Formaldehyde treatment is correlated with significant negative effects on solubility, IgG recovery, and gelling capacity and is also perceived by some customers as a practice to mask low quality raw materials. Moreover, formaldehyde has not proved to be effective against heat and chemical resistant viruses like PPV or PCV-2 [33]. Results obtained in experiment 2 with UV-C irradiated plasma suggested that the microbiological quality of UV-C treated SDPP was improved and that the UV-C irradiation had no negative effect on animal performance. In fact, we observed a tendency of improved pig performance during the first 14 days and better performance over the whole period when pigs were fed diets with UV- irradiated plasma compared with non UV irradiated plasma diets. These results are in agreement with the reported values indicated by DeRouchey et al. [30,31] and Groesbeck et al. [32] using gamma irradiation. Nevertheless, it is worthy to indicate that the control plasma showed absence of *Salmonella* and low level of Enterobacteriaceae indicating the low prevalence of pathogenic microorganisms in commercial SDPP.

## Conclusions

Adaptation of a uniquely designed UV-C process specific for the manufacturing of spray dried plasma that does not adversely affect its beneficial functional components has merit as an additional biosafety feature to protect against unidentified or emerging pathogens that may evolve over time in biological source material. Such redundant technologies are aligned with suggestions from international organizations that recommend biosafety procedures to minimize risks associated with use of biological products.

## Supporting Information

S1 Table Mean PPV virus titers at each irradiation point (PPV titers expressed as log10 TCID50%/ml). (DOC)

S2 Table UV irradiation effect on PPV virus inactivation in bovine plasma. (DOC)

S1 Text Probability of minimum virus titre when no virus is detected in the sample. (DOC)
