# Supplementary material for: Ultraviolet Light (UV) Inactivation of Porcine Parvovirus in Liquid Plasma and Effect of UV Irradiated Spray Dried Porcine Plasma on Performance of Weaned Pigs
Source: PLoS One. 2015 Jul 14;10(7):e0133008. doi: 10.1371/journal.pone.0133008 (PMC4501813; doi:10.1371/journal.pone.0133008)
Supplement: S1 Table — (DOC) [file pone.0133008.s001.doc]

**Table 1. Mean PPV virus titers at each irradiation point (PPV titers expressed as log_10_ TCID50%/ml)**

| Time (min) | UV Irradiation  Doses (J/l) | Mean of 3 replicates | Confidence interval (IC) | IC Max | IC Min | Standard  deviation |
| --- | --- | --- | --- | --- | --- | --- |
| 0 | 0 | 5.20 | 0.13341 | 5.33 | 5.06 | 0.12 |
| 5 | 765 | 1.02 | 0.50306 | 1.53 | 0.52 | 0.44 |
| 10 | 1,530 | 0.15 | 1.11065 | 1.26 | 0.00 | 0.98 |
| 15 | 2,295 | 0.00 | 0.00 | 0.00 | 0.00 | 0.00 |
| 30 | 4,590 | 0.00 | 0.00 | 0.00 | 0.00 | 0.00 |
| 45 | 6,885 | 0.00 | 0.00 | 0.00 | 0.00 | 0.00 |
| 60 | 9,180 | 0.00 | 0.00 | 0.00 | 0.00 | 0.00 |
