# Supplementary material for: Ultraviolet Light (UV) Inactivation of Porcine Parvovirus in Liquid Plasma and Effect of UV Irradiated Spray Dried Porcine Plasma on Performance of Weaned Pigs
Source: PLoS One. 2015 Jul 14;10(7):e0133008. doi: 10.1371/journal.pone.0133008 (PMC4501813; doi:10.1371/journal.pone.0133008)
Supplement: S2 Table — (DOC) [file pone.0133008.s002.doc]

**Table 2.** UV irradiation effect on PPV virus inactivation in bovine plasma

| Experiment replicate | Sample | UV treatment  Time | UV Energy dose Joules/Littre | Virus quantification of plasma samples | | | | | | | Virus detection on falcon with SK6 cells | |
| --- | --- | --- | --- | --- | --- | --- | --- | --- | --- | --- | --- | --- |
|  |  |  |  | Dil -1 | Dil -2 | | Dil -3 | | Dil -4 | | Cell  passage | Results Log 10 DICC50%/mL |
|  | Inoculum | Inoculum | Inoculum |  | |  | |  | |  |  | Titer 7.10 |
|  | Plasma | Plasma | Plasma |  | |  | |  | |  | 3^rd^ | 3º passage negative |
| A | 1 | 0 min | 0 | 48/48^(1)^ | | 48/48 | | 43/48 | | 7/48 | 1^st^ | Titer 5.24±0.13^(2)^ |
| A | 2 | 5min | 120.8 | 0/48 | | 0/48 | | 0/48 | | 0/48 | 2^nd^ | 2º positive, estimated titer <0.16^(3)^ |
| A | 3 | 10min | 241.6 | 0/48 | | 0/48 | | 0/48 | | 0/48 | 3^rd^ | 3º passage negative |
| A | 4 | 15min | 362.4 | 0/48 | | 0/48 | | 0/48 | | 0/48 | 3^rd^ | 3º passage negative |
| A | 5 | 30min | 724.7 | 0/48 | | 0/48 | | 0/48 | | 0/48 | 3^rd^ | 3º passage negative |
| A | 6 | 45min | 1087.1 | 0/48 | | 0/48 | | 0/48 | | 0/48 | 3^rd^ | 3º passage negative |
| A | 7 | 60min | 1449.5 | 0/48 | | 0/48 | | 0/48 | | 0/48 | 3^rd^ | 3º passage negative |
|  | Inoculum | Inoculum | Inoculum |  | |  | |  | |  |  | Titer 7.65 |
|  | Plasma | Plasma | Plasma |  | |  | |  | |  |  | 3º passage negative |
| B | 8 | 0 min | 0 | 48/48 | | 48/48 | | 45/48 | | 10/48 | 1^st^ | Titre 5.35±0.14 |
| B | 9 | 5min | 120.8 | 4/48 | | 4/48 | | 1/48 | | 0/48 | 1^st^ | Estimated titre 2.45±0,34 |
| B | 10 | 10min | 241.6 | 0/48 | | 1/48 | | 1/48 | | 0/48 | 2^nd^ | 2º positive, estimated titer 0.46^(4)^ |
| B | 11 | 15min | 362.4 | 0/48 | | 0/48 | | 0/48 | | 0/48 | 3^rd^ | 3º passage negative |
| B | 12 | 30min | 724.7 | 0/48 | | 0/48 | | 0/48 | | 0/48 | 3^rd^ | 3º passage negative |
| B | 13 | 45min | 1087.1 | 0/48 | | 0/48 | | 0/48 | | 0/48 | 3^rd^ | 3º passage negative |
| B | 14 | 60min | 1449.5 | 0/48 | | 0/48 | | 0/48 | | 0/48 | 3^rd^ | 3º passage negative |
|  | Inoculum | Inoculum | Inoculum |  | |  | |  | |  |  | Titer 7.60 |
|  | Plasma | Plasma | Plasma |  | |  | |  | |  | 3^rd^ | 3º passage negative |
| C | 15 | 0 min | 0 | 48/48 | | 48/48 | | 33/48 | | 4/48 | 1^st^ | Titer 5±0.15 |
| C | 16 | 5min | 120.8 | 1/48 | | 1/48 | | 0/48 | | 0/48 | 1^st^ | 1º positive, estimated titer 0.46^(4)^ |
| C | 17 | 10min | 241.6 | 0/48 | | 0/48 | | 0/48 | | 0/48 | 3^rd^ | 3º passage negative |
| C | 18 | 15min | 362.4 | 0/48 | | 0/48 | | 0/48 | | 0/48 | 3^rd^ | 3º passage negative |
| C | 19 | 30min | 724.7 | 0/48 | | 0/48 | | 0/48 | | 0/48 | 3^rd^ | 3º passage negative |
| C | 20 | 45min | 1087.1 | 0/48 | | 0/48 | | 0/48 | | 0/48 | 3^rd^ | 3º passage negative |
| C | 21 | 60 min | 1449.5 | 0/48 | | 0/48 | | 0/48 | | 0/48 | 3^rd^ | 3º passage negative |

(1) Number of positive wells versus total inoculated wells in 96 wells plates; (2) Spearman Karber ± Standar error (3) At least minimum detection titer if all wells are negative 4 Spearman Karber modification for samples when the lowest dilution when don’t have 100% infected wells.
