# Supplementary material for: Ultraviolet Light (UV) Inactivation of Porcine Parvovirus in Liquid Plasma and Effect of UV Irradiated Spray Dried Porcine Plasma on Performance of Weaned Pigs
Source: PLoS One. 2015 Jul 14;10(7):e0133008. doi: 10.1371/journal.pone.0133008 (PMC4501813; doi:10.1371/journal.pone.0133008)
Supplement: S1 Text — (DOC) [file pone.0133008.s003.doc]

Reduction factor defined as log10 relation between the quantity of virus detected in the started material by unit of volume and the final quantity of virus after final treatment.

Expected clearance factor at 5 min after UV exposition is 10^4.17±0.46^ DICC50%/mL, and expected reduction after 10 min is 10^5.05±0.99^ DICC50%/mL.

**Probability of minimum virus titre when no virus is detected in the sample**

The probability of minimum virus titre after 15 min UV treatment is 0. Considering that treated volume (V) is largest than the analyzed volume (v) in this case a distribution of Poison should be appropriated.

Poison distribution formula could be applied when no virus has been detected in the analized suspension. The formula take in account the tested total volume of sample inoculated to cell culture that will provide the detecction limits of the assay (Remington, 2013. Reference 8 in the text). Then to calculate the probability is necessary to assume that a minimum number of particles (*) could be in one litter of plasma. Total volume tested were 16.2ml.

The formula is:

c = ln p / -v,

where: c = concentration of infectious virus particles in the process intermediate,

p = probability (typically at 95%)

v = the volume of the sample that is actually tested in the assay (by litter).

C result is 225 particles by litter

**Conclusion**

The probability that in a litter would have 225 particles is 0.0479 this mean that in p<0,05% of cases the analyzed final total volume (16.2ml) would not have infectious particles. Then 0.23 (**) particles/ml is a detection limit with a 95% of confidence with the analyzed sample.

Note*: Assuming that one particle could infect and produce a visible cytopathic effect in a falcon bottle of SK6 cells after three blind passages.

A factor of conversion could be used to approximate TCID50% to particles or and “vice versa”:

To convert TCID50%/ml to particles could be transformed dividing by 0.69 or particles to TCID50% multipliying by 0.69 (http://en.wikipedia.org/wiki/Virus_quantification)
